# Role of caspase-3/E-cadherin in helicobacter pylori-induced apoptosis of gastric epithelial cells

**DOI:** 10.18632/oncotarget.19471

**Published:** 2017-07-22

**Authors:** Yongmei Yang, Jie Du, Fen Liu, Xiaoyan Wang, Xiaohui Li, Yuanjian Li

**Affiliations:** ^1^ Department of Pharmacology, Xiangya School of Pharmaceutical Science, Central South University, Changsha, China; ^2^ Department of Anatomy, School of Medicine, University of South China, Hengyang, China; ^3^ Department of Gastroenterology, Third Xiangya Hospital, Central South University, Changsha, China

**Keywords:** helicobacter pylori, caspase-3, E-cadherin, E-cadherin/carboxy-terminal fragment 3 (E-cad/CTF3), apoptosis

## Abstract

This study was designed to investigate the role of caspase-3/E-cadherin in *Helicobacter pylori* (*H. pylori*) -induced gastric epithelial apoptosis in cells, animal models and clinical gastritis patients. In cultured gastric mucosal epithelial cells, gastric glandular epithelial cells and C57BL/6 mice, *H. pylori* infection significantly induced apoptosis of gastric epithelial cells, down-regulated full-length E-cadherin and Bcl-2 expression, and up-regulated cleaved-caspase-3, E-cadherin/carboxy-terminal fragment 3 and Bax expression. Z-DEVD-FMK, an inhibitor of caspase-3, attenuated the effect of *H. pylori*. E-cadherin overexpression significantly inhibited the apoptosis of GES-1 and SGC-7901 cells induced by the *H. pylori*. The results from clinical studies also showed down-regulation of E-cadherin, up-regulation of cleaved-caspase-3 expression and increased apoptosis in gastric tissues from gastritis patients with *H. pylori* infection. These results suggest that the caspase-3/E-cadherin pathway is involved in the apoptosis of gastric epithelial cells induced by *H. pylori*.

## INTRODUCTION

Peptic ulcer is characterized by the formation of chronic gastric ulcer or duodenal mucosa ulcer, and the prevalence varies from 11% to 69% in different countries [1]. Peptic ulcer can be complicated by bleeding, perforation, pyloric obstruction and inducing cancer. The incidence of this disease involves a variety of factors, such as dietary, chemical (smoking, alcohol and drugs), biological (*Helicobacter pylori, H. pylori*), mental and environmental factors. Among them, *H. pylori* is considered as an important pathogenic factor [2]. However, the mechanism of *H. pylori*-induced gastric mucosal injury has not been fully elucidated.

*H. pylori*-induced gastric epithelial cell apoptosis is a major mechanism for gastric mucosal damage. *in vitro* and *in vivo* experiments showed that *H. pylori* and the secretion of cytotoxins, such as cytotoxin-associated protein A (CagA), vacuolating cytotoxin A (VacA), lipopolysaccharide, and others, could induce gastric epithelial cell apoptosis and gastric ulcer formation [3, 4]. Previous studies have shown that cell apoptosis was related to caspase-3 pathway activation [5, 6], which was partly attributed to mitochondrial outer membrane permeability changes [7–9]. Caspase-3 induces apoptosis by cleaving specific substrates, which has been documented by the generation of caspase-3 cleaved substrates, accompanied by morphological and molecular characteristics of apoptosis [10, 11].

Recently, caspase-3 was shown to cleave E-cadherin, which is believed to be an important activation mechanism in cellular apoptosis [12]. E-cadherin is a single transmembrane glycoprotein with a molecular weight of approximately 120 kDa, which is predominantly distributed in epithelial cells to maintain the integrity of cell-cell binding through calcium-dependent homodimers [13, 14]. E-cadherin is involved a variety of cellular biological functions, such as cell proliferation [15], differentiation and apoptosis [16–18]. E-cadherin consists of three parts: ① a long extracellular region with N-terminal sugar chains is recognized by the ligand region, ② the transmembrane region is a α-helix that penetrates the membrane, and ③ the short cytoplasmic region comprises the C-terminal portion of the peptide chain, which can associate with the cytoskeletal component of the plasma membrane or intracellular signal transduction proteins (α-catenin or β-catenin) and then connect the actin filaments [16, 19, 20]. Studies have shown that E-cadherin/carboxy-terminal fragment 3 (E-cad/CTF3) is an intracellular fragment of E-cadherin that stimulates apoptosis in the human breast epithelial cell line H184A1 and MDCK (Madin-Darby canine kidney) cells [12]. Based on the effect of *H. pylori* on cleavage of E-cadherin and activation of caspase-3 [7, 21], we hypothesized that *H. pylori* induces apoptosis of gastric epithelial cells by activation of caspase-3, which cleaves E-cadherin to produce intracellular fragment 3, resulting in gastric mucosal injury.

In the present study, the effect of caspase-3/E-cadherin on cellular apoptosis induced by *H. pylori* was investigated in cultured gastric mucosal epithelial cells (GES-1) and gastric glandular epithelial cells (SGC-7901). Furthermore, in C57BL/6 mice, the effect of the caspase-3 inhibitor Z-DEVD-FMK on gastric mucosal injury induced by *H. pylori* was tested to confirm the role of caspase-3/E-cadherin. Finally, the relationship between caspase-3/E-cadherin expression and gastric mucosal lesions was assessed in *H. pylori*-infected gastritis patients.

## RESULTS

### Effect of *H. pylori* on apoptosis of gastric epithelial cells

**Figure 1:**
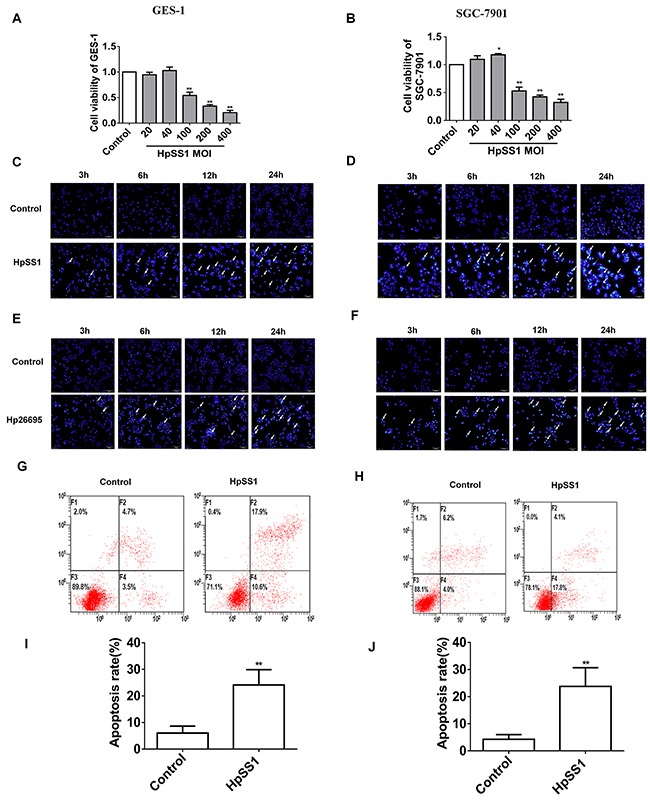
Effect of *H. pylori* on apoptosis of gastric epithelial cells **(A and B)** Gastric epithelial cell viability. **(C-J)** Gastric epithelial cell apoptosis. Data are expressed as the mean ± standard error. n=3, **P < 0.01 vs. Control.

**Figure 2:**
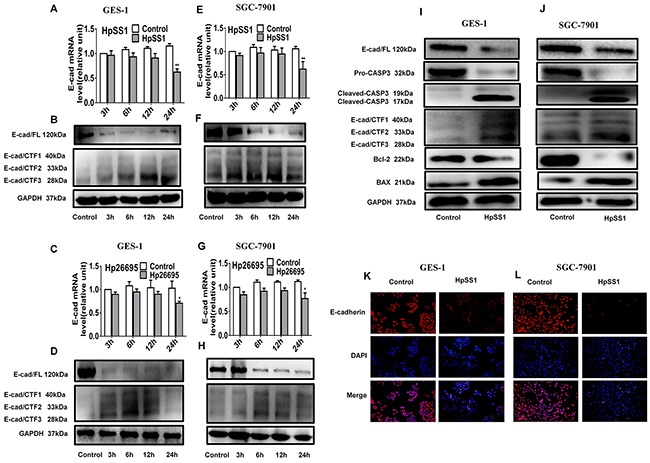
Effects of *H. pylori* on activation of caspase-3 and cleavage of E-cadherin in GES-1 and SGC-7901 cells **(A-D)** E-cadherin and the intracellular fragments of E-cadherin expression in GES-1 cells. **(E-H)** E-cadherin and the intracellular fragments of E-cadherin expression in SGC-7901 cells. **(I)** The expression of pro-caspase-3, cleaved-caspase-3, E-cadherin, E-cadherin intracellular fragments and apoptosis-related proteins in GES-1 cells. **(J)** The expression of pro-caspase-3, cleaved-caspase-3, E-cadherin, E-cadherin intracellular fragments and apoptosis-related proteins in SGC-7901 cells. **(K and L)** E-cadherin expression in gastric epithelial cells. Each experiment was performed in triplicate and repeated at least three times, and representative graphs are provided.

GES-1 and SGC-7901 cell lines were incubated with different concentrations of HpSS1 [multiplicity of infection (MOI) 0, 20, 40, 100, 200, 400]. When the MOI was more than 100, the cell viability was significantly decreased, and the higher the multiplicity of infection, the lower the cell viability. In the follow-up study, we selected an HpSS1 MOI of 100 as the injury concentration (Figure 1A, 1B). *H. pylori* infection induced significant cellular apoptosis (Figure 1C-1F, 1G-1J, Supplementary Figure 1A-1D). Bcl-2 expression was down-regulated, and Bax expression was up-regulated in epithelial cells incubated with *H. pylori* (Figure 2I, 2J).

### Effects of *H. pylori* on activation of caspase-3 and cleavage of E-cadherin in GES-1 and SGC-7901 cells

To investigate the changes of caspase-3/E-cadherin in *H. pylori*-induced gastric epithelial cell apoptosis, the caspase-3 expression and cleavage of E-cadherin in GES-1 and SGC-7901 cells were assessed. The results showed that the protein level of cleaved-caspase-3 was significantly up-regulated by HpSS1 infection for 6 h, indicating activation of caspase-3 (Figure 2I, 2J). The mRNA expression of E-cadherin was also significantly down-regulated by HpSS1 and Hp26695 after a 24 h treatment. The protein expression of E-cadherin was down-regulated at 6 h, 12 h and 24 h, while the levels of E-cadherin/carboxy-terminal fragment 1 (E-cad/CTF1), E-cadherin/carboxy-terminal fragment 2 (E-cad/CTF2) and E-cad/CTF3 were up-regulated, and E-cad/CTF3 expression was significantly increased at 6 h and 12 h (Figure 2A-2H). These results indicated that both HpSS1 and Hp26695 could effectively cleave E-cadherin, producing cytoplasmic fragments to induce apoptosis, and HpSS1 showed more power to cleave E-cadherin at12 h and 24 h. In the follow-up mechanistic study, we conducted experiments using HpSS1 as a stimulator. Immunofluorescence detection showed significant down-regulation of E-cadherin expression in HpSS1-induced GES-1 and SGC-7901 cells (Figure 2K, 2L), which was consistent with the results from western blotting.

### Effects of E-cadherin overexpression on apoptosis of GES-1 and SGC-7901 cells induced by *H. pylori*

**Figure 3:**
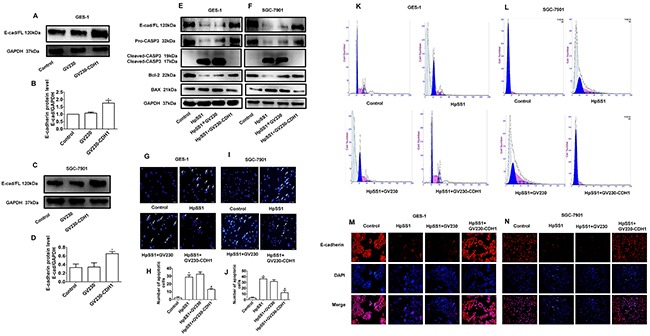
Effects of E-cadherin overexpression on apoptosis of GES-1 and SGC-7901 cells induced by H. pylori **(A-D)** Overexpression of the GV230-CDH1 plasmid. The data are expressed as the mean ± standard error, n=3, *P < 0.05 vs. Control. **(E)** The expression of pro-caspase-3, cleaved-caspase-3, E-cadherin and apoptosis-related proteins in GES-1 cells. **(F)** The expression of pro-caspase-3, cleaved-caspase-3, E-cadherin and apoptosis-related proteins in SGC-7901 cells. **(G-J)** Apoptosis of gastric epithelial cells. Data are expressed as the mean ± standard error, n=8, **P < 0.01 vs. Control, ^#^*P* < 0.05 vs. HpSS1+GV230. **(K and L)** Apoptosis of gastric epithelial cells. **(M and N)** E-cadherin expression in gastric epithelial cells. Each experiment was performed in triplicate and repeated at least three times, and representative graphs are provided.

To investigate the effect of E-cadherin on the apoptosis of GES-1 and SGC-7901 cells induced by HpSS1, E-cadherin was transfected in GES-1 and SGC-7901 cells (Supplementary Figure 2A, 2B). Overexpression of E-cadherin was confirmed by western blotting and immunofluorescence (Figure 3A-3D, 3M, 3N). The results showed that overexpression of GV230-CDH1 significantly up-regulated pro-caspase-3 and Bcl-2 expression and down-regulated cleaved-caspase-3 and Bax expression (Figure 3E, 3F). Overexpression of GV230-CDH1 significantly reduced the number of G0 phase cells (Figure 3K, 3L) and inhibited the apoptosis of GES-1 and SGC-7901 cells induced by HpSS1 (Figure 3G-3J). The results of immunofluorescence analysis showed that overexpression of GV230-CDH1 inhibited the down-regulation of E-cadherin expression in GES-1 and SGC-7901 cells induced by HpSS1 (Figure 3M, 3N).

### Effects of the caspase-3 inhibitor Z-DEVD-FMK on E-cad/CTF3 generation and cellular apoptosis induced by *H. pylori*

**Figure 4:**
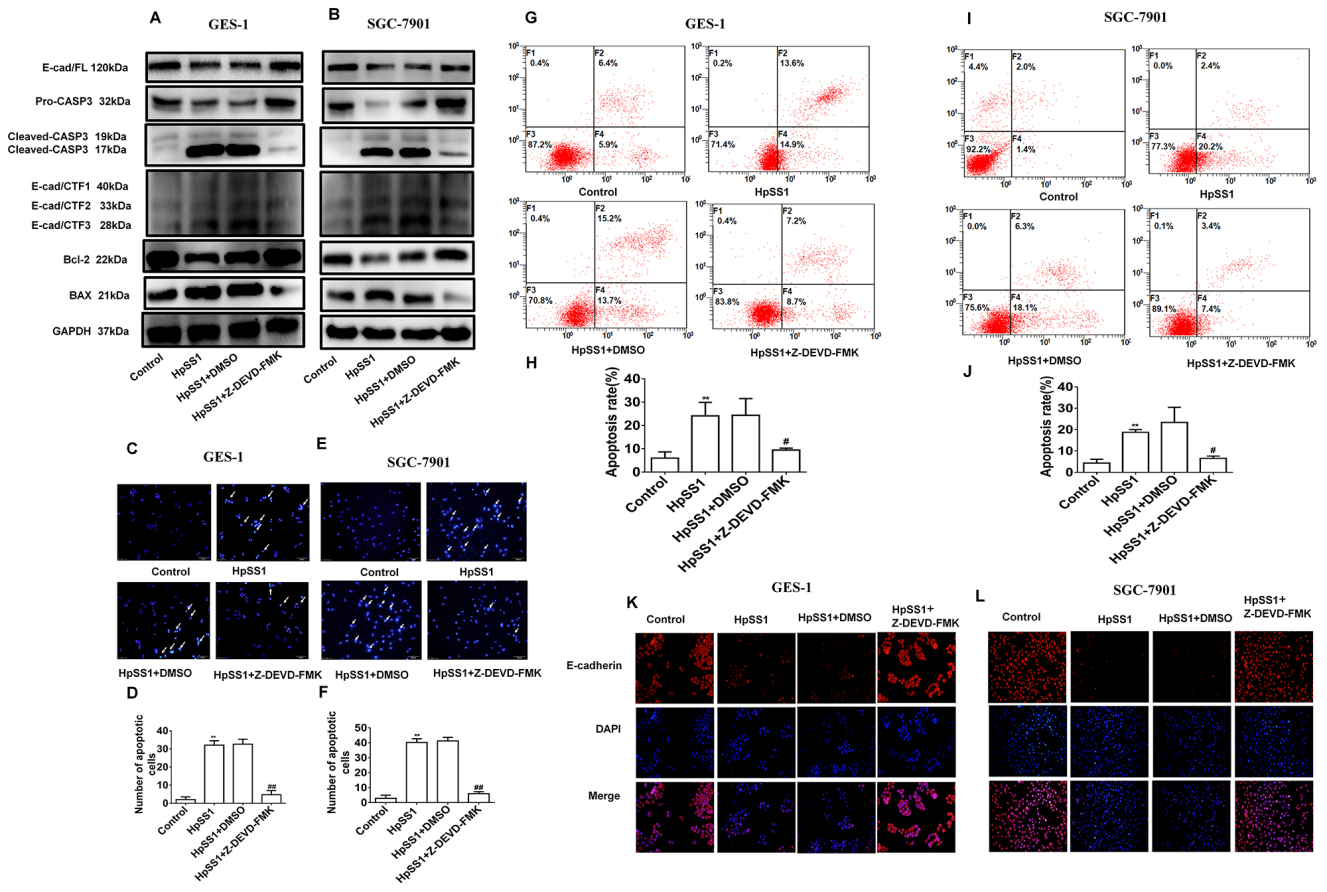
Effects of the caspase-3 inhibitor Z-DEVD-FMK on E-cad/CTF3 generation and cellular apoptosis induced by *H. pylori* **(A)** The expression of pro-caspase-3, cleaved-caspase-3, E-cadherin, E-cadherin intracellular fragments and apoptosis-related proteins in GES-1 cells. **(B)** The expression of pro-caspase-3, cleaved-caspase-3, E-cadherin, E-cadherin intracellular fragments and apoptosis-related proteins in SGC-7901 cells. **(C-F)** Apoptosis of gastric epithelial cells. Data are expressed as the mean ± standard error, n=8, **P < 0.01 vs. Control, ^##^*P* < 0.01 vs. HpSS1+DMSO. **(G-J)** Apoptosis of gastric epithelial cells. Data are expressed as the mean ± standard error. n=3, **P < 0.01 vs. Control, ^#^*P* < 0.05 vs. HpSS1+DMSO. **(K and L)** The expression of E-cadherin in gastric epithelial cells. Each experiment was performed in triplicate and repeated at least three times, and representative graphs are provided.

To investigate the role of caspase-3 and E-cad/CTF3 in gastric epithelial cell apoptosis, the caspase-3 inhibitor Z-DEVD-FMK was utilized. The results showed that Z-DEVD-FMK abrogated the cleavage of E-cadherin by inhibiting caspase-3 activation, as E-cad/CTF3 and cleaved-caspase-3 were significantly reduced (Figure 4A, 4B). Notably Z-DEVD-FMK had no significant effect on E-cad/CTF1 and E-cad/CTF2 generation. Meanwhile, Z-DEVD-FMK induced up-regulation of Bcl-2 and down-regulation of Bax expression in cells treated with HpSS1 for 6 h (Figure 4A, 4B). The results of Annexin V-FITC flow cytometry and Hoechst staining showed that Z-DEVD-FMK significantly inhibited the apoptosis of GES-1 and SGC-7901 cells induced by HpSS1 (Figure 4C-4J). Immunofluorescence analysis indicated that Z-DEVD-FMK attenuated the down-regulation of E-cadherin expression in GES-1 and SGC-7901 cells treated with HpSS1 (Figure 4K, 4L).

### Effects of the caspase-3 inhibitor Z-DEVD-FMK on E-cad/CTF3 production and gastric mucosal injury in C57BL/6 mice induced by *H. pylori*

**Figure 5:**
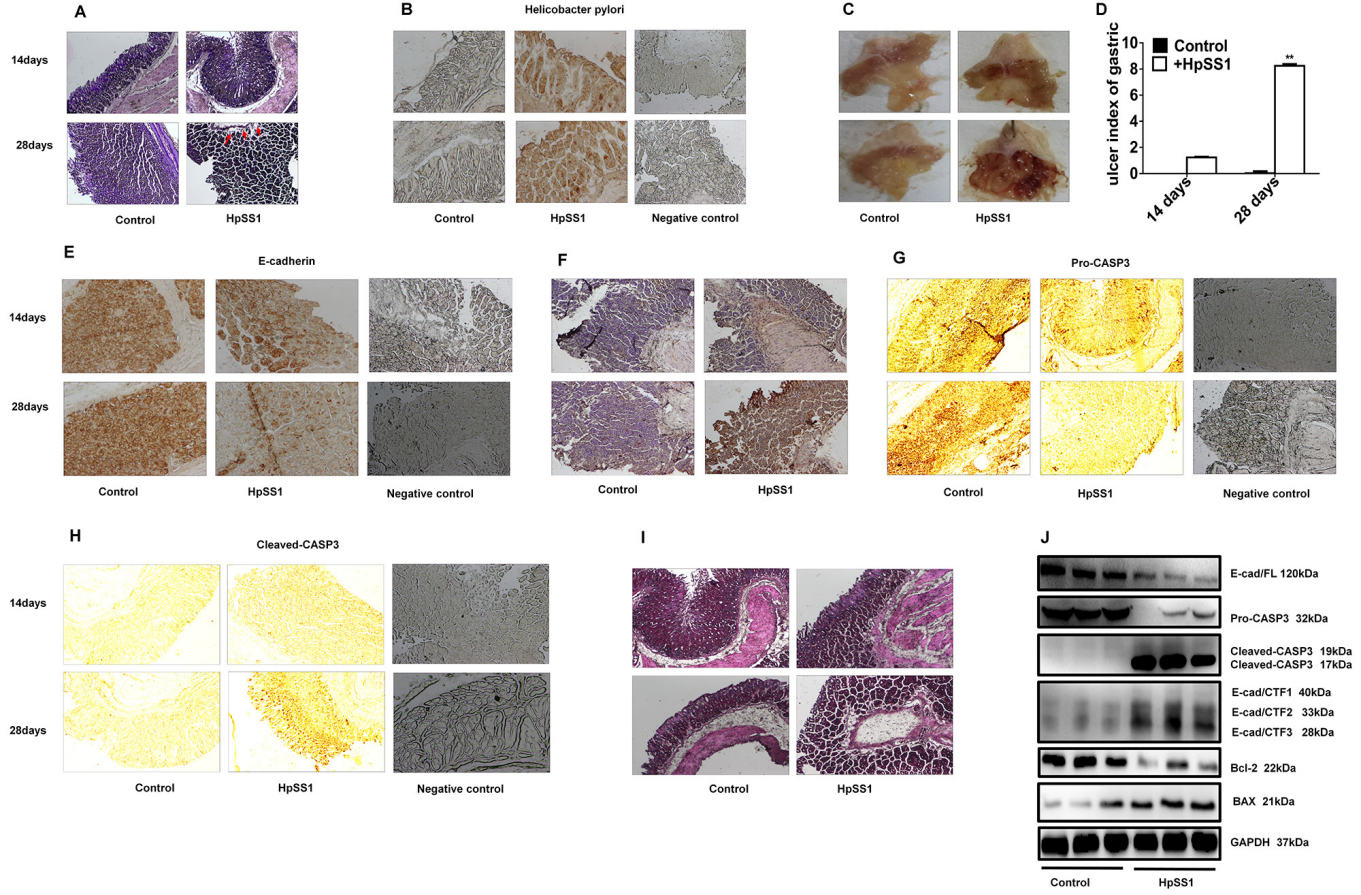
Effects of *H. pylori* on E-cad/CTF3 production and gastric mucosal injury in C57BL/6 mice **(A and B)** Infection of *H. pylori* in gastric mucosa. **(C and D)** Gastric mucosal lesion. Data are expressed as the mean ± standard error, n = 10. **P < 0.01 vs. Control, 28 days. **(E)** E-cadherin expression. **(F)** Cell apoptosis. **(G)** Pro-caspase-3 expression. **(H)** Cleaved-caspase-3 expression. **(I)** Changes in morphology. **(J)** The expression of pro-caspase-3, cleaved-caspase-3, E-cadherin, E-cadherin intracellular fragments and apoptosis-related proteins in gastric mucosa of mice. Each experiment was performed in triplicate and repeated at least three times, and representative graphs are provided.

**Figure 6:**
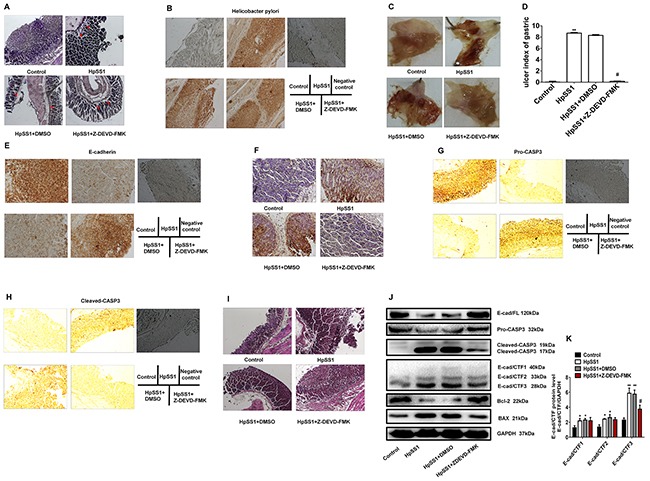
Effects of the caspase-3 inhibitor Z-DEVD-FMK on E-cad/CTF3 production and gastric mucosa injury in C57BL/6 mice induced by *H. pylori* **(A and B)** Infection of *H. pylori* in gastric mucosa. **(C and D)** Effects of Z-DEVD-FMK on HpSS1-induced gastric mucosal injury in mice. Data are expressed as the mean ± standard error, n=10. **P < 0.01 vs. Control; ^#^*P* < 0.05 vs. HpSS1+DMSO. **(E)** E-cadherin expression. **(F)** Cell apoptosis. **(G)** Pro-caspase-3 expression. **(H)** Cleaved-caspase-3 expression. **(I)** Changes in morphology. **(J and K)** Effects of Z-DEVD-FMK on the expression of pro-caspase-3, cleaved-caspase-3, E-cadherin, E-cadherin intracellular fragments and apoptosis-related proteins in gastric mucosa of mice induced by HpSS1. Data are expressed as the mean ± standard error, n=3. *P < 0.05, **P < 0.01 vs. Control; ^#^*P* < 0.05 vs. HpSS1+DMSO.

C57BL/6 mice were treated continuously with HpSS1 for 28 days (2×10^9^/mouse/day). *H. pylori* infection was observed in the gastric mucosa of C57BL/6 mice (Figure 5A, 5B). HpSS1 infection induced gastric mucosal injury in mice, as the gastric ulcer index was increased significantly (Figure 5C, 5D). Z-DEVD-FMK did not diminish HpSS1 infection in mice but significantly reversed the gastric mucosal injury induced by HpSS1, as shown by the decreased gastric ulcer index (Figure 6A-6D). Apoptosis of gastric epithelial cells was significantly increased by HpSS1 infection (Figure 5F), accompanied by down-regulation of E-cadherin and pro-caspase-3 expression (Figure 5E, 5G), up-regulation of cleaved-caspase-3 expression (Figure 5H), and alteration of cellular morphology (Figure 5I). These changes were significantly abolished by Z-DEVD-FMK treatment (Figure 6E-6I). HpSS1 infection significantly induced down-regulation of Bcl-2 and up-regulation of Bax expression (Figure 5J), which were inhibited by Z-DEVD-FMK (Figure 6J). At the same time, E-cad/CTF1, E-cad/CTF2 and E-cad/CTF3 were up-regulated by HpSS1 infection (Figure 5J), and Z-DEVD-FMK treatment reduced E-cad/CTF3 generation but showed no significant effects on the production of E-cad/CTF1 and E-cad/CTF2 (Figure 6J, 6K).

### Effects of *H. pylori* on expression of E-cadherin and cleaved-caspase-3 and apoptosis of gastric tissues in gastritis patients

**Figure 7:**
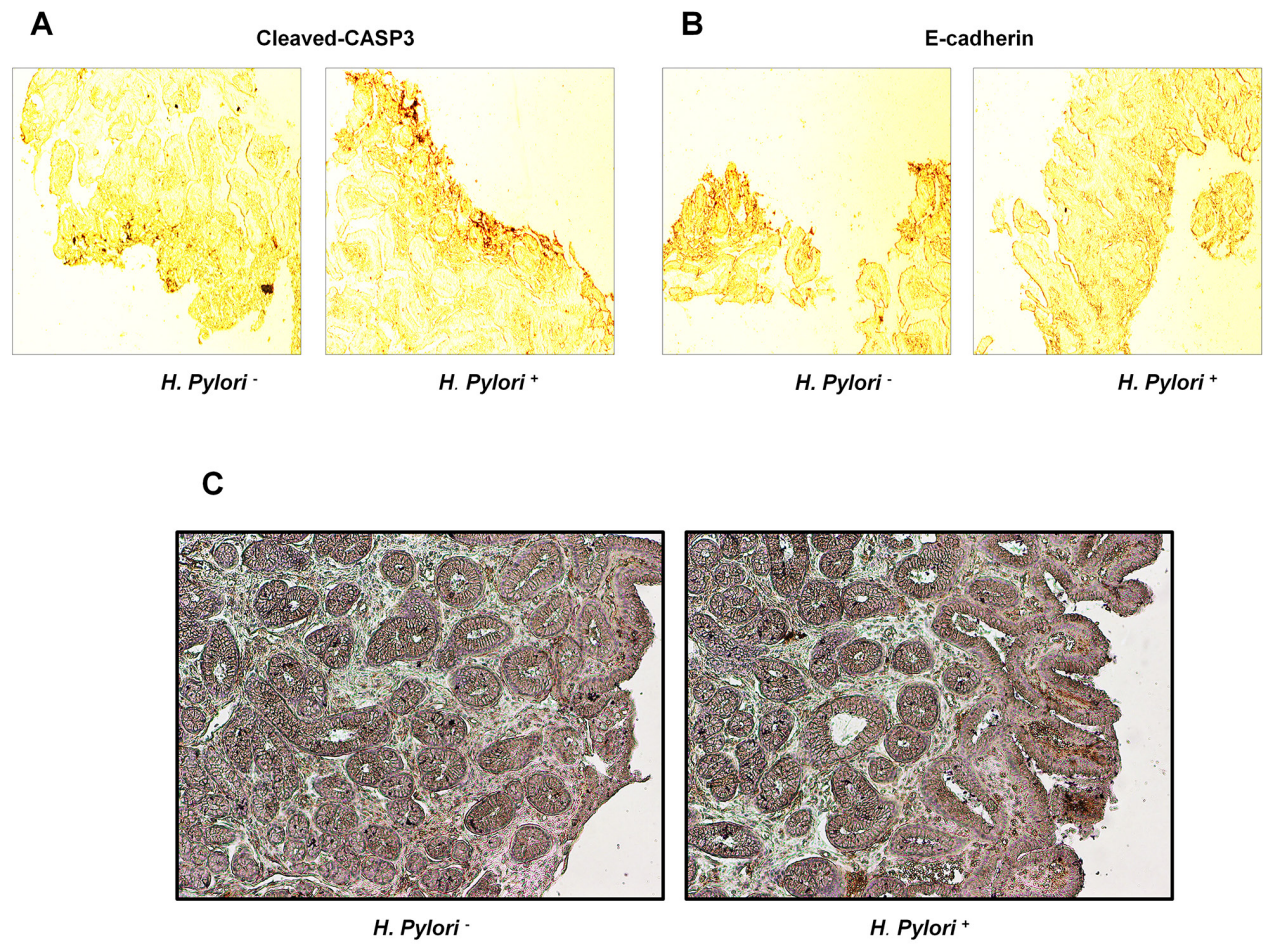
Effects of *H. pylori* on expression of E-cadherin, cleaved-caspase-3 and apoptosis of gastric tissues in gastritis patients **(A)** Cleaved-caspase-3 expression. **(B)** E-cadherin expression. **(C)** Cell apoptosis.

Gastric tissue specimens were collected from 40 gastritis patients aged 23 to 66 years in the Third Xiangya Hospital, Central South University; there were 21 cases of *H. pylori*-negative patients and 19 cases of positive patients. Immunohistochemical staining showed that the expression of cleaved-caspase-3 was significantly elevated and E-cadherin was down-regulated in patients with *H. pylori* infection (Table 1, Figure 7A, 7B), and gastric epithelial apoptosis was significantly increased by *H. pylori* infection (Figure 7C).

**Table 1:** The patient's basic information

	Gastritis patients without *H. pylori* infection	Gastritis patients with *H. pylori* infection1
Number of patients	21	19
male patients	10	9
female patients	11	10
age range	23 to 65 years old	27 to 66 years old
average age	44.76 years old	48.32 years old

## DISCUSSION

*H. pylori* infection plays a critical role in the pathogenesis of chronic gastritis and gastric ulcer diseases, and gastric epithelial apoptosis is an important pathophysiological hallmark of gastric mucosal injury [22, 23]. The mechanism of *H. pylori*-induced epithelial cell apoptosis has not been fully elucidated. Studies have shown that *H. pylori* can induce gastric mucosal injury by increasing expression of Fas and facilitating the interaction of Fas and Fas ligand (FasL) in gastric epithelial cells [24, 25]. *H. pylori* is also able to trigger apoptosis through bacterial toxins [26, 27]. For example, VacA, a pore-forming toxin, induced gastric mucosal injury by initiating Cl^-^ influx, attacking mitochondria, triggering pre-inflammatory factor releasing, and decreasing intracellular ATP levels [28–30]. Others have reported that non-phosphorylated CagA could interact with E-cadherin to form CagA/E-cadherin complex to damage the gastric mucosa [31, 32].

In the present study, we confirmed previous observations that *H. pylori* infection induces gastric epithelial cell apoptosis with up-regulation of cleaved caspase-3 expression and down-regulation of E-cadherin expression and extended these observations to provide evidence that *H. pylori* induces gastric epithelial cell apoptosis by cleavage of E-cadherin to form the E-cad/CTF3 fragment via activation of caspase-3.

E-cadherin is a single transmembrane glyco-protein with a molecular weight of approximately 120 kDa. It is an intercellular connexin protein that binds to the homologous adhesion molecules on the adjacent cell surface via calcium-dependent homodimers [13, 14]. In a variety of tumor cells, E-cadherin has been shown to be closely related with apoptosis [12, 33, 34]. In squamous epithelial cells and primary proximal tubular epithelial cells, E-cadherin could maintain cell and extracellular matrix anchorage to prevent apoptosis, as inhibition of E-cadherin function increased cell apoptosis [35, 36]. The present experiments showed that *H. pylori* infection induced down-regulation of E-cadherin expression accompanied by significant apoptosis *in vivo* and *in vitro*. Moreover, overexpression of E-cadherin significantly reduced apoptosis induced by *H. pylori* in both GES-1 and SGC-7901 cells. These results support the hypothesis that *H. pylori*-induced apoptosis is involved in the E-cadherin pathway. However, the mechanism by which E-cadherin inhibits apoptosis is still unknown. Previous studies have shown that E-cadherin was involved in the phosphatidylinositol 3 kinase (PI-3K) signaling pathway by increasing PI-3K-dependent survival in renal tubular epithelial cells [35]. In the present study, *H. pylori*-induced cellular apoptosis, down-regulation of Bcl-2 expression and up-regulation of Bax expression were reversed by overexpression of E-cadherin. Recently, it has been demonstrated that *H. pylori* VacA could trigger mitochondria-dependent apoptosis of gastric epithelial cells through the down-regulation of cellular Bcl-2 and the up-regulation of Bax [37, 38]. These findings prompted us to hypothesize that E-cadherin-mediated apoptosis may affect apoptosis-related gene expression.

Activation of a specific class of proteolytic enzymes in cells is the most typical molecular change in apoptosis; these enzymes cleave a variety of intracellular proteins (such as cytoskeletal proteins, signal molecules, and others) and activate intracellular nucleases to cleave DNA, inducing apoptosis. Caspase-3, an analog of CED-3, is a proteolytic enzyme associated with apoptosis in nematode cells. Caspase generally stimulates apoptosis by cleaving specific substrates. Caspase-3 induced apoptosis by cleaving E-cadherin in the human breast epithelial cell line H184A1 and MDCK (Madin-Darby canine kidney) cells [12], suggesting that E-cadherin is a substrate of caspase-3. This study confirmed that *H. pylori* infection up-regulated cleaved caspase-3 expression accompanied by down-regulation of full-length E-cadherin expression and enhanced cleavage of E-cadherin in gastric epithelial cells and mice. E-cadherin is cleaved to produce various intracellular fragments due to different enzymes, different cells and different treatment factors. The results of this study showed that *H. pylori* (HpSS1 and Hp26695) caused generation of three intracellular fragments (E-cad/CTF1, E-cad/CTF2, E-cad/CTF3) by cleavage of E-cadherin in SGC-7901 and GES-1 cells, accompanied by gastric epithelial cell apoptosis. These results suggest that *H. pylori*-induced apoptosis is related to the formation of the intracellular domain of E-cadherin. In the present study, the expression of the intracellular domain was up-regulated significantly at 6 h and 12 h and decreased after 24 h, which may be related to degradation of the protein. Pretreatment with the caspase-3 inhibitor Z-DEVD-FMK almost completely blocked formation of the E-cad/CTF3 fragment stimulated by staurosporine (STS) but had no significant effect on the generation of other intracellular fragments [12]. In the present experiment, Z-DEVD-FMK was utilized in the *H. pylori* infection model. The results showed that the production of the intracellular E-cad/CTF3 (28 kDa) fragment was significantly decreased, while caspase-3 activity was inhibited. All these findings suggest that E-cad/CTF3, an important intracellular fragment of E-cadherin cleaved by caspase-3, induces apoptosis. As described above, E-cad/CTF1 and E-cad/CTF2 were also produced in gastric epithelial cells treated with *H. pylori* in addition to E-cad/CTF3. E-cad/CTF1 is the residual part of E-cadherin cleaved by a matrix metalloproteinase (MMP) in the human breast epithelial cell line H184A1, MDCK (Madin-Darby canine kidney) cells and A431 epidermal cancer cells [12, 39]. This study also found that E-cad/CTF1 was produced in SGC-7901 and GES-1 cells infected with *H. pylori*, which may be related to activation of MMPs. The size of E-cad/CTF2 was consistent with the intracellular fragment of E-cadherin cleaved by high-temperature requirement A (HtrA), a *H. pylori* pathogenic factor reported previously [21]. The function of E-cad/CTF2 is to destroy the gastric epithelial cell adhesion, guiding the bacteria into the cell gap. In the present study, E-cad/CTF1 and E-cad/CTF2 were generated by *H. pylori* treatment and were not decreased by a caspase-3 inhibitor. However, the role of these E-cadherin fragments and cleavage enzymes in *H. pylori*-induced gastric injury requires further investigation.

Apoptosis involves two major pathways, in which activation of caspase-8 usually occurs as a result of Fas binding to its ligand. Activation of the Fas-Fas ligand system was involved in *H. pylori*-associated apoptosis *in vivo* and *in vitro* [25], and a caspase-8 inhibitor was reported to inhibit *H. pylori*-induced apoptosis [40]. The present study showed that *H. pylori* induced apoptosis with down-regulation of Bcl-2 expression and up-regulation of Bax and E-cad/CTF3 expression, which were all abolished by the caspase-3 inhibitor Z-DEVD-FMK. These results suggested that the two major pathways of apoptosis, the Fas/Fas ligand system and the mitochondrial pathway, are involved in *H. pylori-*induced apoptosis of gastric epithelial cells.

In summary, this experiment showed that *H. pylori* infection induced gastric epithelial apoptosis through activation of caspase-3 and cleavage of E-cadherin. Therapy targeting the caspase-3/E-cadherin pathway may have potential future clinical applications for *H. pylori* infection.

## MATERIALS AND METHODS

### Cell culture and transfections

The human gastric mucosal epithelial cell line GES-1 was obtained from the Advanced Research Center, Central South University in China. The human gastric glandular epithelial cell line SGC-7901 was a gift from the Cancer Research Institute, University of South China. Cell lines were grown in Dulbecco's modified Eagle's medium (Gibco) containing 10% fetal calf serum (HyClone) in a humidified atmosphere at 37°C and 5% CO_2_. Cells were seeded in tissue culture plates before infection and grown to confluence. For inhibitor studies, cells were preincubated for 30 min with 50 μM Z-DEVD-FMK before addition of HpSS1. For overexpression experiments, cells were transfected with GV230-CDH1 or GV230 empty plasmid before addition of HpSS1 using FECT™ CP Transfection Kit (RiboBio) for 24 h according to the manufacturer's instructions.

### Clinical subjects

The scientific research project approval to conduct this study was obtained from The Third Xiangya Hospital, Hunan, China (approval number: 2015-S109). A total of 40 patients with gastritis were enrolled in the clinical study, including 21 *H. pylori*-negative peptic ulcer cases and 19 *H. pylori*-positive cases. Inclusion criteria of patients for clinical study were as follows: gastric histology was determined with a gastroscope, antral biopsies were taken for urease tests, and pathology and culture were performed for analysis of *H. pylori* colonization. Expression of cleaved-caspase-3 and E-cadherin was determined by immunohistochemical analysis of the samples. Apoptosis of gastric epithelial cells was detected by terminal deoxynucleotidyl transferase-mediated dUTP-biotin nick end labeling (TUNEL) staining.

### Animal models and specimen collection

All animal experiments and procedures were approved by the Animal Care Committee at the Department of Laboratory Animals, Central South University (approval number: (SCKK(su)2011-0003). C57BL/6 male mice (8 weeks old, 19 to 22 g) were randomly divided into four groups (n=10): ① the control group was perfused with phosphate-buffered saline (PBS) daily, ② the injury group was treated with HpSS1 dissolved in PBS, 2 ×10^9^/mouse/day, ③ HpSS1 + solvent (DMSO < 0.5%) group: after 30 min of intraperitoneal injection, the mice were perfused with HpSS1, ④ HpSS1 + Z-DVED-FMK group: after 30 min of drug treatment (intraperitoneal injection, 1.5 mg/kg/d), the mice were perfused with HpSS1. After 4 weeks of HpSS1 infection, gastric tissue of the mice was separated, cut along the large curvature of the stomach, washed and photographed, and the bleeding areas were counted. Samples of gastric tissue proteins were extracted for western blotting assays. A portion of the gastric tissue was fixed in 4% paraformaldehyde solution for morphological analysis.

### Bacteria and infection experiments

The *H. pylori* strain 26695 was purchased from the American Type Culture Collection (ATCC). The *H. pylori* strain SS1 was obtained from the Institute of Pathogenic Biology, University of South China. Hp26695 and HpSS1 were cultured on agar plates containing 10% sheep blood under microaerophilic conditions at 37°C. For infection, bacteria were harvested in PBS (pH 7.4) and added to the host cells at a MOI of 100.

### Induction of apoptosis and preparation of cell lysates

Apoptosis was induced in confluent monolayers of cells cultured in 6-well dishes by addition of the *H. pylori* strains Hp26695 or HpSS1 at a MOI of 100. Adherent cells were washed twice with PBS and scraped off from the culture dish. Floating cells were collected by gentle centrifugation at 1000 rpm for 5 min and were incubated with RIPA Lysis Buffer (50 mM Tris (pH 7.4), 150 mM NaCl, 1% Triton X-100, 1% sodium deoxycholate, 0.1% SDS, sodium orthovanadate, sodium fluoride, EDTA, leupeptin (Beyotime Biotechnology)) for 30 min on ice. After centrifugation (15 min, 4 °C, 12,000 rpm), the supernatant was collected for western blotting. Total protein concentration of the cell lysates was determined using a BCA protein assay kit (Beyotime Biotechnology)

### SDS-PAGE and western blots

For western blotting, 50 μg of total protein in 5×SDS loading buffer was separated on 10% ProSieve SDS-polyacrylamide gels. Primary antibodies against E-cadherin (1:1000), pro-caspase-3 (1:400), caspase-3 (active 1:120), Bcl-2 (1:600), and Bax (1:500) and the secondary antibodies HRP-anti-mouse (1:5000) and HRP-anti-rabbit (1:5000) were used. The relative optical density of each band was densitometrically analyzed, and the results are expressed as the ratio of normalized to the GAPDH levels. The Bio-Rad ChemiDoc XRS + imaging system was used for photographic analysis, and Image Lab software was utilized for gray value analysis.

### Assessment of apoptosis

Apoptosis was induced in 5×10^5^ cells seeded into 6-well dishes by addition of *H. pylori* strains at a MOI of 100 and was measured by flow cytometry, using an Annexin V-FITC Apoptosis Detection Kit and Cell Cycle and Apoptosis Analysis Kit (Beyotime Biotechnology) according to the manufacturer's protocol. The apoptotic cells were stained with Hoechst 33258 (Beyotime Biotechnology) and were assessed under an inverted fluorescence microscope (Japan Nikon company) according to the manufacturer's guidelines. Apoptotic cells in C57BL/6 mouse stomach tissue sections were evaluated by the terminal deoxynucleotidyl transferase-mediated dUTP-biotin nick end labeling (TUNEL) assay using an In Situ Cell Death Detection Kit, POD (Roche) following the manufacturer's instructions.

### RNA extraction and real-time RT-PCR

Total RNA was extracted (TaKaRa, Japan) according to the manufacturer's instructions, and 500 ng RNA was reverse transcribed using a reverse transcription-PCR (RT-PCR) kit according to the manufacturer's protocol (TaKaRa, Japan). The expression of E-cadherin mRNA (*Forward: 5’- TTGCTACTGGAACAGGGACAC -3’; Reverse: 5’-CCCGTGTGT TAGTTCTGCTGT -3’*) was measured using an ABI 7300 real-time PCR system (Applied Biosystems, Foster City, Calif., USA) with the SYBR Green PCR Master Mix (TOYOBO, Japan). GAPDH was used as an internal control in quantitative analysis. The gene expression level was normalized to GAPDH and expressed as the ratio of E-cadherin to GAPDH mRNA.

### Immunofluorescence analysis

Cells were rinsed with PBS and fixed with 4% paraformaldehyde for 15 min at room temperature. After washing 3 times in PBS, cell permeabilization was performed with 0.25% Triton X-100 in PBS at room temperature for 10 min. The cells were blocking with 1% BSA at 37°C for 1 h and rinsed three times with PBS before incubated with anti-E-cadherin (Clone 36/E-Cadherin 1:50 dilution BD Transduction Laboratories™) antibody at 4°C overnight. A donkey anti-mouse secondary antibody conjugated to Cy3 was incubated for 1 h before immunofluorescence detection. Nuclei were stained with DAPI for 1 min. Images were acquired using an immunofluorescence microscope (Olympus) equipped with a 20 ×objective, analyzed and processed by a Nikon & spot image acquisition system.

### Immunohistochemistry

For immunohistochemistry, sections from C57BL/6 mice and 21 *H. pylori*^-^ and 19 *H. pylori*^+^ gastritis patient gastric mucosa biopsies were deparaffinized and rehydrated, followed by antigen retrieval using citrate buffer (pH 6.1) for 20 min and were then stained with primary antibodies overnight at 4°C. Slides were washed and treated with the universal type SP kit and DAB kit (ZSGB-BIO) according to the manufacturer's instructions. Images were acquired using an immunofluorescence microscope (Olympus) equipped with a 20×objective, analyzed and processed by a Nikon & spot image acquisition system.

### Hematoxylin-eosin staining

Paraffin sections were deparaffinized and placed in double distilled water for 10 minutes, followed by hematoxylin-eosin (HE) staining according to the manufacturer's instructions.

### Statistical analysis

All data are expressed as the mean ± standard error. Multiple mean comparison was performed by ANOVA and Student-Newman-Keuls multiple comparison t tests. Statistical analysis was performed using SPSS 17.0 software, and *P* < 0.05 was considered statistically significant.

### Reagents and antibodies

Caspase-3 inhibitor II (Z-DEVD-FMK) was purchased from Calbiochem. Terminal deoxynucleotidyl transferase mediated dUTP nick end labeling (TUNEL) was purchased from Roche. SYBR Green real-time polymerase chain reaction (PCR) master mix was purchased from TaKaRa (Shiga, Japan). The universal type SP kit and DAB kit were purchased from ZSGB-BIO. The Annexin V-FITC apoptosis detection kit and the cell cycle and apoptosis detection kit were purchased from Beyotime Biotechnology. The plasmids (GV230 and GV230-CDH1) were designed and synthesized by Shanghai Genechem Co., Ltd. The monoclonal antibody directed against the E-cadherin cytoplasmic domain (clone 36) was purchased from Transduction Laboratories (Lexington, KY). The polyclonal antibodies directed against *H. pylori* were purchased from Abcam (ab187301). The polyclonal antibodies directed against pro-caspase-3, caspase-3 (active), Bcl-2, and Bax were purchased from Sangon Biotech. The monoclonal antibody directed against GAPDH was purchased from Beyotime Biotechnology. HRP-anti-mouse antibody and HRP-anti-rabbit antibody were purchased from Boster Biological Technology. Cy3-labeled donkey anti-mouse antibody was purchased from Sangon Biotech.

## SUPPLEMENTARY MATERIALS FIGURES



## Abbreviations

E-cad/CTF1: E-cadherin/carboxy-terminal fragment 1
E-cad/CTF2: E-cadherin/carboxy-terminal fragment 2
E-cad/CTF3: E-cadherin/carboxy-terminal fragment 3
*H. pylori*: *Helicobacter pylori*
HpSS1: *H. pylori* strain SS1
Hp26695: *H. pylori* strain 26695
pro-CASP3: pro-caspase-3
cleaved-CASP3: cleaved-caspase-3
GES-1: gastric mucosal epithelial cells
SGC-7901: gastric glandular epithelial cells
E-cad/FL: full-length E-cadherin
CagA: cytotoxin associated protein A
VacA: Vacuolating cytotoxin A
MDCK: Madin-Darby canine kidney
MOI: multiplicity of infection
PI-3K: phosphatidylinositol 3 kinase
STS: staurosporine
MMP: matrix metall-oproteinase
HtrA: high-temperature requirement A
TUNEL assay: terminal deoxynucleotidyl transferase-mediated dUTP-biotin nick end labeling assay
HE: hematoxylin-eosin
PBS: phosphate-buffered saline
