# Supplementary material for: Role of caspase-3/E-cadherin in helicobacter pylori-induced apoptosis of gastric epithelial cells
Source: Oncotarget. 2017 Jul 22;8(35):59204–16. doi: 10.18632/oncotarget.19471 (PMC5601725; doi:10.18632/oncotarget.19471)
Supplement: Supplementary file 1 [file oncotarget-08-59204-s001.pdf]

Role of caspase-3/E-cadherin in helicobacter pylori-induced apoptosis of gastric epithelial cells

SUPPLEMENTARY MATERIALS

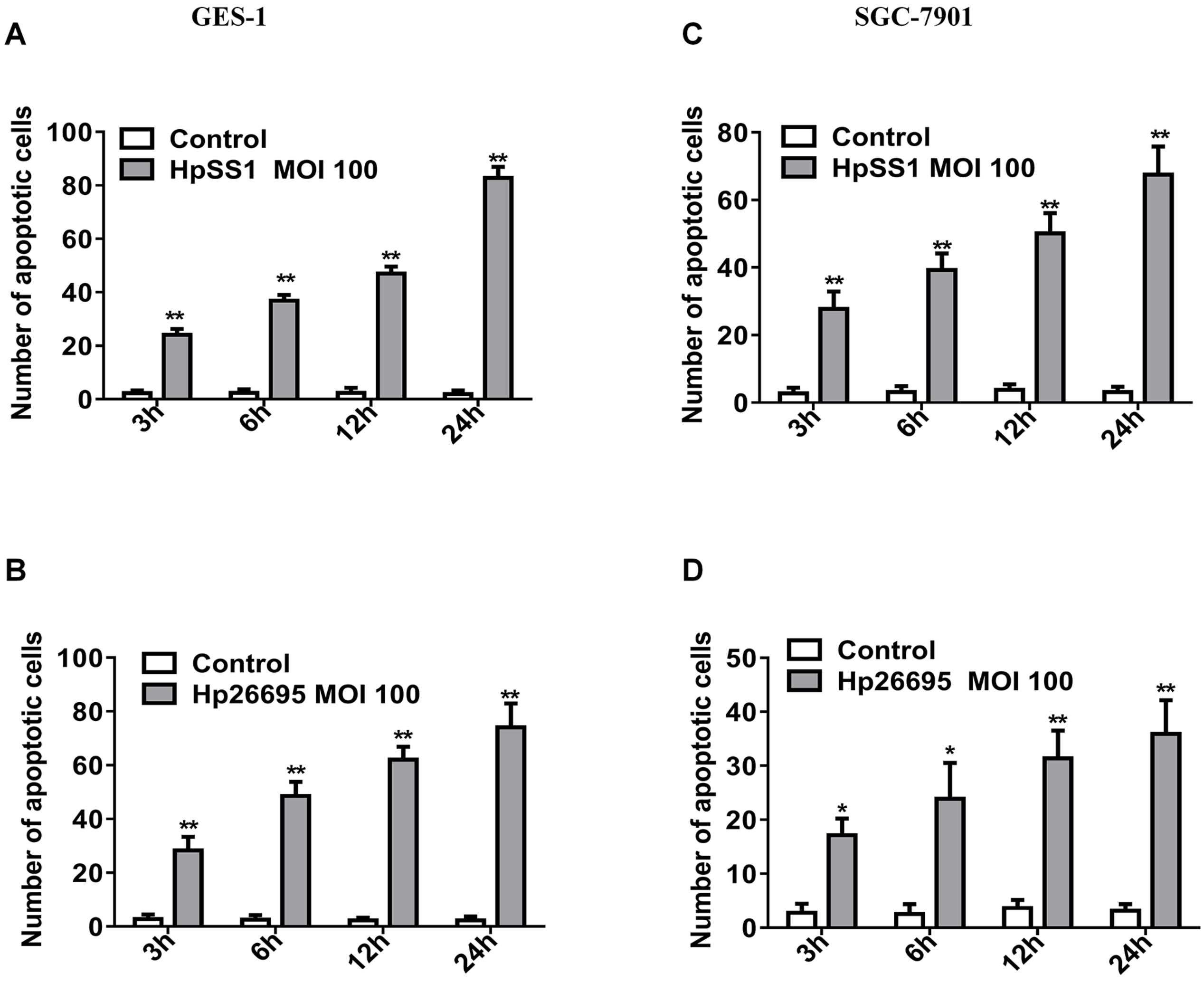

Supplementary Figure 1: Detection of apoptosis in gastric epithelial cells by Hoechst staining. (A-D) Number of apoptotic gastric epithelial cells induced by H. pylori in GES-1 or SGC-7901. Data are expressed as the mean ± standard error, n = 8, **P < 0.01 vs. Control at different time points; *P < 0.05 vs. Control 3 h, 6 h; **P < 0.01 vs. Control 12 h, 24 h.

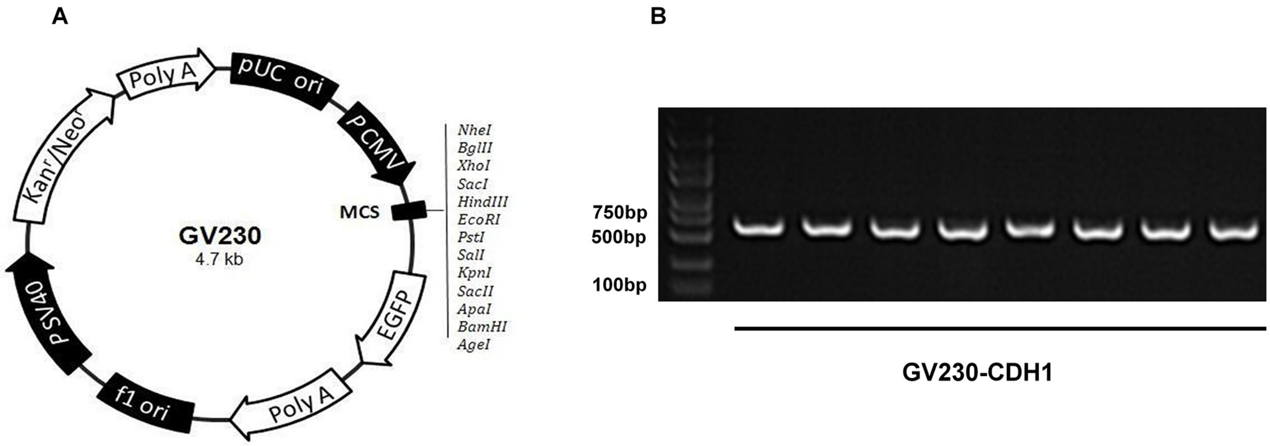

Supplementary Figure 2: Mapping and identification of the GV230-CDH1 plasmid. (A) Mapping of the GV230-CDH1 plasmid. **(B)** Identification of the GV230-CDH1 plasmid.
